# Coinfection of some respiratory viruses in cattle: An abattoir study

**DOI:** 10.4102/ojvr.v91i1.2193

**Published:** 2024-12-06

**Authors:** Intisar K. Saeed, Yahia H. Ali, Muaz Magzoub Abdellatif, Alaa Mustafa, Ahmad M. Abdel-Mageed

**Affiliations:** 1 Department of Biology, College of Science and Arts, Northern Border University, Arar, Saudi Arabia; 2 Virology Department, Central Veterinary Research Laboratory, Khartoum, Sudan; 3 Department of Biological Sciences, College of Science, Northern Border University, Arar, Saudi Arabia; 4 Department of Mathematics, College of Science and Arts, Northern Border University, Arar, Saudi Arabia; 5 Zoology Department, Faculty of Science, Minia University, Minia, Egypt

**Keywords:** Cattle, BVD, PIV-3, RSV, coinfection

## Abstract

To investigate the existence of bovine viral diarrhoea virus (BVDV), parainfluenza virus 3 (PIV-3) and respiratory syncytial virus (RSV) as well as its coinfections, a total of 420 pneumonic lung tissue samples were collected from slaughterhouses in three different areas. Samples were examined for the three viruses using antigen detection enzyme-linked immunosorbent assay (ELISA) test, and positive results were further confirmed using fluorescent antibody test and polymerase chain reaction. Prevalences detected were 10.47% for BVDV, 11.9% for parainfluenza virus type (PIV-3) and 7.6% for respiratory syncytial virus (RSV). Observed coinfections were bovine viral diarrhoea (BVD) and PIV-3 in 8.1%, BVD and RSV in only two samples (1%). Existence of all three viruses was detected in two samples (1%).

**Contribution:**

In this study, infection of the three viruses was confirmed in cattle and existence of its coinfection is documented for the first time.

## Introduction

Bovine respiratory disease complex (BRDC) was found to be a major cause of death in cattle (Dorso et al. 2021). It causes significant economic losses in cattle, beef and dairy production, and is caused by different viral and bacterial pathogens (Werid et al. 2024a). Viruses encountered in the syndrome include bovine respiratory syncytial virus (BRSV), bovine parainfluenza virus 3 (BPIV-3), bovine viral diarrhoea virus 1 (BVDV-1) (Werid et al. 2024a). Globally, BVDV, parainfluenza virus 3 (PIV-3), respiratory syncytial virus (RSV) are the most common viruses found to be encountered in many respiratory infections. In China, BVDV antibodies in dairy were 90% while for beef cattle it was 63% (Deng et al. 2015). In Japan, BVD cases are increasing (Tajima 2021). Existence of BVD in cattle was reviewed in Indonesia (Nugroho et al. 2022), Iran (Khodakaram-Tafti & Farjanikish 2017) and Mexico (Gomez-Romero et al. 2021). Also in, Columbia (González-Bautista et al. 2021), Ireland (Barrett et al. 2024), Spain (Nodar et al. 2023), Switzerland (Schweizer et al. 2021), Poland (Socha et al. 2022). The prevalence of BVDV1 in North America is observed to be increasing (Walz et al. 2020). Similar findings were reported in Africa, for example, Ethiopia (Yitagesu et al. 2021), Kenya (Okumu et al. 2019; Olum et al. 2020) and Sudan (Intisar et al. 2015b).

Parainfluenza virus 3 is known to cause respiratory infections in cattle. Bovine parainfluenza virus 3 is a negative-stranded ribonucleic acid (RNA) enveloped virus in the bovine respirovirus genus in the family Paramyxoviridae (Adams et al. 2017). Bovine parainfluenza virus 3 was found to be the predominant single agent for respiratory infection in cattle in Switzerland (Mehinagic et al. 2019) and China (Leal et al. 2019), and a survey on BPIV-3 in Egypt showed a higher rate of infection in cattle than in buffaloes (Sobhy et al. 2017). The virus was detected in cattle in France and Sweden (Gaudino et al. 2023), in Tatarstan (Gueriche et al. 2020) and the existence of BPIV-3 infection in cattle in 32 countries was reviewed (Werid et al. 2024b). In Sudan, the association of the virus with respiratory infections in cattle was reported previously (Intisar et al. 2016; Noori et al. 2014, 2018).

Bovine respiratory syncytial virus is one of the major causes of BRDC; it belongs to the Pneumovirus genus of the family Paramyxoviridae (Yaman et al. 2018). Respiratory syncytial virus antibodies were found in cattle in India (Goswami et al. 2016). In China, a disease outbreak with 27% morbidity and more than 25% mortality rates was detected in beef cattle calves (Jia et al. 2021) and 19% positivity of RSV was reported (Chang, Yue & Tang 2022). Bovine respiratory syncytial virus was reported in Iraq (Hussain et al. 2019), Turkey (Yazici et al. 2020), Italy (Giammarioli et al. 2020), Norway (Klem, Rimstad & Stokstad 2014), Argentina (Ferella et al. 2023), Egypt (Zaher et al. 2014) and Sudan (Intisar 2019).

Respiratory viral infections in cattle may be caused by a single virus or multiple viruses which usually exaggerate the syndrome. Many reports documented the occurrence of viral respiratory coinfection. Mixed infections with PIV-3, RSV, BVD, bovine herpes virus type 1 (BHV-1) and/or some bacterial species were more frequent (72%) than single infections in dairy calves in Brazil (Oliveira et al. 2020) and, in dual purpose cattle in Colombia (León et al. 2019). Similar results were observed in India (Gangil, Kaur & Dwivedi 2020), China (Zhou et al. 2023), Saudi Arabia (Ali & Gomaa 2014; Mahmoud & Allam 2013), Kenya (Callaby et al. 2016) and North America (Fulton 2020).

This study is intended to explore the recent prevalence of BVD, PIV-3, RSV as well as the existence of viral coinfection in viral respiratory infected cattle in Sudan.

## Research methods and design

### Sample collection

**FIGURE 1:**
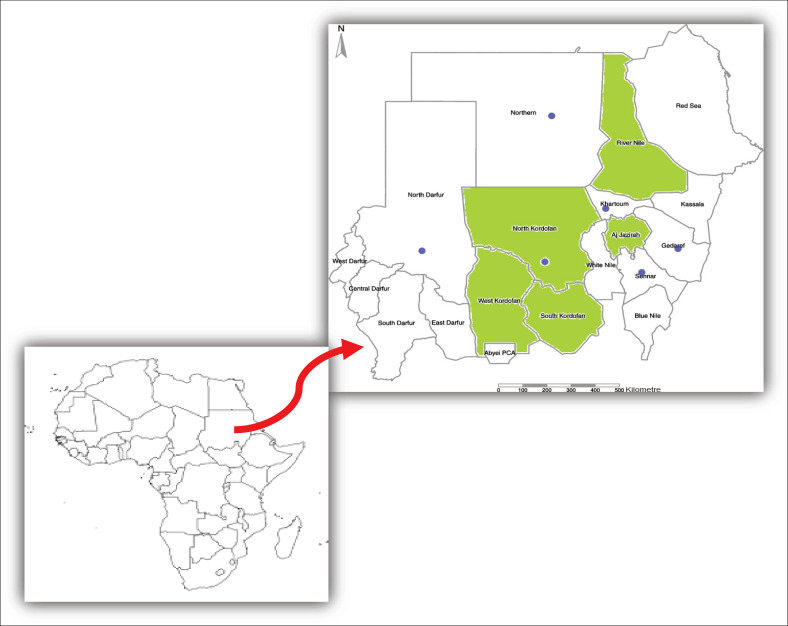
Map of Sudan showing states where samples were collected.

Pneumonic lung tissue samples (*n* = 420) were collected from slaughterhouses in three regions of Sudan, Atbara at River Nile State (*n* = 180), Wad Medani at Gezira State (*n* = 80), and 160 samples from AlObied at Kordofan State (Figure 1). Samples were sent on ice to the Central Veterinary Research Laboratory in Khartoum and kept frozen until they were investigated.

### Serological investigation

#### Bovine viral diarrhoea virus antigen detection

Tissues were examined for BVDV antigen using ELISA (enzyme-linked immunosorbent assay) kits obtained from BIO X Diagnostics (Jemelle, Belgium) following the directions provided by the manufacturer.

### Fluorescent antibody technique for detection of bovine viral diarrhoea virus antigen

The ELISA-positive samples were tested for BVDV antigen using fluorescent antibody test (FAT) Kits provided by BIO X Diagnostics (Jemelle, Belgium).

### Parainfluenza virus 3 antigen detection

Samples were tested for PIV3 antigen using ELISA kits (BIO X Diagnostics, Jemelle, Belgium) as instructed by the manufacturer.

### Fluorescent antibody test for detection of parainfluenza virus 3 antigen

The ELISA reactive samples were tested for PIV3 antigen using FAT (BIO X Diagnostics, Jemelle, Belgium).

### Respiratory syncytial virus antigen detection

Lung tissue samples were tested for the detection of RSV antigen using ELISA kits from BIO X Diagnostics, Jemelle, Belgium, following the manufacturer instructions.

### Fluorescent antibody test for respiratory syncytial virus antigen detection

The ELISA-positive samples were screened for RSV antigen using FAT; the conjugate was purchased from BIO X Diagnostics, Jemelle, Belgium.

### Molecular investigation

#### Polymerase chain reaction

Polymerase chain reaction (PCR) was carried out on tissues (*n* = 6) randomly picked from ELISA-positive results for each virus tested.

#### Ribonucleic acid extraction

Lung tissue suspensions (*n* = 6) were used for purification of the virus nucleic acids as described by Chomcznki and Sacchi (1989). The method was done using 0.2 g of crushed tissues which were frozen at −80 °C in phenol and guanidine isothiocyanate (TRIzol Reagent; Gibco-BRL [Bethesda Research Laboratories]). Then incubated at room temperature for 5 min, and 250 µL of chloroform was added. After vigorous shaking for 2 min the tubes were centrifuged (12 000 × g for 15 min at 4 °C) and left to stand at room temperature for 10 min. One millilitre of phenol and guanidine isothiocyanate (TRIzol LS Reagent; Gibco BRL) was added to the supernatant in each tube. Precipitation of RNA was done in isopropanol (volume/volume [vol/vol]) with 0.3 M sodium acetate (pH 5.2) (Sigma) overnight at 4 °C. Centrifuged (12 000 × g for 10 min at 4 °C), and the pellet was washed in 1 mL of 70% ethanol, then centrifuged (7500 × g for 5 min at 4 °C), the pellet was dried for 10 min and the RNA was resuspended in 36 µL of diethyl pyro carbonate-treated (DEPC) water, kept at 4 °C till used.

#### Reverse transcription-polymerase chain reaction for identification of bovine viral diarrhoea virus

Amplification was done using one step reverse transcription-polymerase chain reaction (RT/PCR) Kits (Qiagen), using the following primers; 5′- CATGCCCWYAGTAGGACTAGC-3′ and 5′- AACTCCATGTGCCATGTACAG-3′ (Becher, Orlich & Thiel 1998). The conditions were reverse transcription at 50 °C for 30 min followed by 94 °C for 15 min; thermocycling for 40 cycles at 94 °C for 30 s, 55 °C for 30 s and 72 °C for 30 s; one cycle at 72 °C for 10 min.

#### Reverse transcription-polymerase chain reaction for detection of parainfluenza virus 3

Primers targeting HA gene were selected (PI3 A 5′-TGTGCATGGTGAGTTCGCA-3′, PI3 BR 5′-ATTCAGCATCACGTGCCACTG-3′), and the expected product size is 164 base pairs (bp) (Noori et al. 2014). Ribonucleic acid template (5 µL) was added into 45 µL master mix. Amplification conditions were reverse transcription in one step RT/PCR kit reagents at 50 °C for 30 min followed by 94 °C for 15 min. Then, 40 cycles of PCR, denaturation at 94 °C for 30 s, annealing at 56 °C for 30 s, elongation of 72 °C for 30 s, and final extension at 72 °C for 10 min.

#### Reverse transcription-polymerase chain reaction for detection of respiratory syncytial virus

Reverse transcription-polymerase chain reaction for RSV RNA was applied according to Samal et al. (1991) in two rounds. Primers used in the first round were primer 1(5-ATGGCTCTCAGCAAGGTCA-3; positions 1–19 on the nucleocapsid (N) coding region of the bovine RSV genome) and primer 2 (50 pmol) (5-TCTTGGTTTCTTGGTGTACCTC-3; positions 1034–1013 on the N coding region of the BRSV genome) (Samal et al. 1991). The programme applied was 94 °C for 12 min, 35 cycles of denaturation at 94 °C for 60 s, annealing at 58 °C for 60 s and elongation at 72 °C for 90 s. For the second PCR round, 10 µL of the PCR product diluted 10 times was used with the same mix containing the internal primers 3 (5-CATCTCAATAAGTTGTGTGG-3); positions 127–146 of the N coding region of the BRSV genome and 4 (5-TCTACAACCTGTTCCATTTC-3; positions 857–838 on the N coding region of the RSV genome) (Samal et al. 1991). Programme used for the second round of PCR which amplified 731 nucleotides (nt): was 94 °C for 12 min, 35 cycles of denaturation at 94 °C for 45 s, annealing at 49 °C for 60 s and elongation at 72 °C for 60 s and ending with a final elongation for 10 min.

### Gel electrophoresis

The amplified products were documented on a 1.5% agarose (Vivantis) gel using a horizontal mini-gel electrophoresis system (MSMINI, Cleaver Scientific). Deoxyribonucleic acid (DNA) bands were visualised using a gel documentation system (Ingenius, Syngene Bio Imaging).

### Statistical analysis

The ELISA results were analysed to estimate the frequency of BVD, PIV-3, RSV infections, and to examine the occurrence of mixed BVD, PIV-3, RSV virus infections. Chi-square of independence was adopted to test the null hypothesis of association between BVD, location, PIV3 and RSV (*p* < 0.001) using SPSS 27 (Statistical Package for Social Sciences 27; IBM, SPSS, Chicago, Illinois, United States).

### Ethical considerations

This article followed all ethical standards for research without direct contact with human or animal subjects.

## Results

### Serological findings

#### Detection of bovine viral diarrhoea virus antigen

**FIGURE 2:**
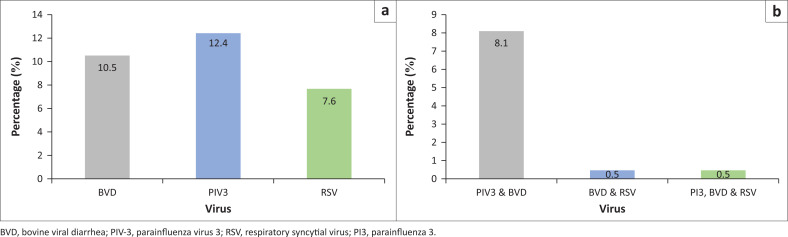
Percentage of the overall percentage of seropositivity according to virus (a) and mixed infection (b) of bovine viral diarrhoea, parainfluenza virus 3 and respiratory syncytial virus.

Out of 420 lung tissues examined, 10.5% tested positive, the highest prevalence (15.6%) was seen in River Nile State (Table 1; Figure 2).

**TABLE 1:** Frequency of virus infection according to location.

Location	BVD	PIV-3	RSV
**River Nile**			
Count	28	40	28
%	15.6	22.2	15.6
**Algazeira**			
Count	10	12	0
%	12.5	15.0	0.0
**Kordofan**			
Count	6	0	4
%	3.8	0.0	2.5

BVD, bovine viral diarrhoea; PIV-3, parainfluenza virus 3; RSV, respiratory syncytial virus.

### Fluorescent antibody technique

The ELISA-positive samples (*n* = 44) were found to be positive using FAT.

### Parainfluenza virus 3 antigen detection

The overall prevalence of PIV-3 detected was 12.4%, with the highest prevalence (22.2%), found in River Nile State (Table 1; Figure 2).

### Fluorescent antibody test for detection of parainfluenza virus 3 antigen

Parainfluenza virus 3 ELISA-positive samples (*n* = 52) examined by FAT showed positive results.

### Respiratory syncytial virus antigen detection

Out of 420 lung tissue samples examined for RSV antigen detection, 7.6% were tested positive, and the highest prevalence (26.7%) was detected in River Nile State (Table 1; Figure 2).

### Fluorescent antibody test for respiratory syncytial virus antigen detection

All ELISA-positive samples (*n* = 52) for RSV antigen showed positive result using FAT.

### Coinfection of parainfluenza virus 3, respiratory syncytial virus, bovine viral diarrhoea virus viruses

All samples (*n* = 420) were examined for the BVD, PIV-3 and RSV antigen using ELISA. Coinfections of BVDV and PIV-3 were observed in 34 samples (8.1%), the highest prevalence (12.2%) was found in River Nile State. Coinfection of BVDV and RSV was observed in only two samples in River Nile State (1.1%). Existence of all three viruses, BVDV, PIV-3 and RSV was detected in two samples (1.1%) also in River Nile State (Table 2; Figure 2).

**TABLE 2:** Frequency of coinfection according to location.

Location	PIV3 & BVD	BVD & RSV	PI3, BVD & RSV
**River Nile**			
Count	22	2	2
%	12.2	1.1	1.1
**Algazeira**			
Count	8	0	0
%	10.0	0.0	0.0
**Kordofan**			
Count	4	0	0
%	2.5	0.0	0.0

BVD, bovine viral diarrhoea; PIV-3, parainfluenza virus 3; RSV, respiratory syncytial virus; PI3, parainfluenza 3.

### Molecular findings

#### Bovine viral diarrhoea virus nucleic acid detection

**FIGURE 3:**
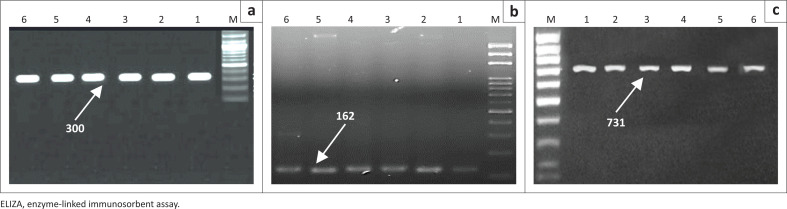
Ethidium bromide-stained agarose gel. Reverse transcription-polymerase chain reaction was carried out on ribonucleic acid purified from lung tissues using specific primers for bovine viral diarrhoea (a), parainfluenza virus 3 (b) and respiratory syncytial virus (c). Lane M, 100 base pairs molecular weight marker, lane 1-6, ELISA-positive samples.

All selected ELISA-positive samples tested for BVDV nucleic acid showed positive results with amplicon size of 300 bp (Figure 3).

### Parainfluenza virus 3 nucleic acid detection using reverse transcription-polymerase chain reaction

Selected PIV-3 ELISA-positive samples examined for nucleic acid detection showed positive results with product size of 164 bp (Figure 3).

### Respiratory syncytial virus nucleic acid detection

Applied RT/PCR for RSV nucleic acid on selected ELISA-positive samples showed positive results with a band size of 731 bp (Figure 3).

### Chi-square test

Statistical analysis indicates significant association between BVDV and PIV3 (*p* = 0.000), BVDV and RSV (*p* = 0.000) (Table 3).

**TABLE 3:** Correlation of bovine viral diarrhoea, location, parainfluenza virus 3 and respiratory syncytial virus, showing the Pearson chi-square value and *p*-value.

Parameter	Location	PIV3	RSV
Pearson Chi-square	6.510†	123.991†	82.496†
*df*	2.000	1.000	1.000
Asymptotic significance	0.039	< 0.000	< 0.000

*df*, degree of freedom.

† , Statistically significant if *p*-value < 0.05.

## Discussion

Respiratory infections in cattle are major constraints of animal production, causing significant economic losses because of deaths and decreased productivity. In this study, through antigen detection tools, BVDV prevalence of 10.5% in cattle was found, concurring with the result reported in Spain (Nodar et al. 2023) and very close (10.7%) to that obtained previously in Sudan (Intisar et al. 2015b). However, the highest prevalence in the previous study was observed in Central Sudan. Our results are higher than those reported in different countries, for example, China 1% (Deng et al. 2015; Su et al. 2023), Bangladesh 3% (Alam et al. 2016), 2% in Brazil (Freitas et al. 2021) and Japan (Tajima 2021). The prevalence of BVDV was reviewed from 2010 to 2021 globally in 128 reports, and it was 16%, with the lowest prevalence (0.3%) reported in North America (Su et al. 2023).

Parainfluenza virus 3 is one of the known viral causes of BRDC. In this study, the overall prevalence of PIV-3 detected was 12%, and the highest prevalence (21%) was found in River Nile State. Previously, almost the same result (13%) was reported in Sudan, where highest prevalence was detected in Gezira at Central Sudan (Intisar et al. 2016). However, higher prevalence (20%) was detected in Kordofan at Western Sudan (Noori et al. 2014). The results of both reports in Sudan are higher than those reported in several countries, for example, Brazil 3% (De Oliveira et al. 2022) and 8% (Oliveira et al. 2021), India 5% (Gangil et al. 2020) and China 6% (Guo et al. 2021) and 7% (Veljović et al. 2016). A slightly higher prevalence (14%) was reported in Brazil (Oliveira et al. 2020) and a much higher prevalence (38%) was reported in Switzerland (Mehinagic et al. 2019).

Bovine respiratory syncytial virus was documented to have a significant role in causing respiratory infections in cattle. The detected prevalence of RSV in this study was 7.6%, the highest prevalence (14%), was detected in River Nile State like the other viruses. This is lower than that (13%) previously reported in Sudan (Intisar 2019); nevertheless, it is higher than 0.6% detected prevalences in Italy (Padalino et al. 2021), 6% in China (Zhou et al. 2023) and Turkey (Yaman et al. 2018). Similar and comparable prevalences were detected in Mongolia (7%) (Guo et al. 2021), in the United States (9%) (Fulton 2020) and in Turkey (11%) (Aydin et al. 2024). Higher prevalences were reported in Argentina (14%) (Ferella et al. 2023), China (25% – 30%) (Jia et al. 2021), Norway (49%) (Klem et al. 2014) and Poland (60%) (Urban-Chmiel et al. 2015), while the highest prevalence (100%) was reported in an outbreak of lethal respiratory disease in Italy (Giammarioli et al. 2020).

Bovine respiratory disease complex is most probably caused by two or more viruses, sometimes associated with bacterial pathogen which exaggerate the syndrome. Coinfection with more than one virus is known to exist in many BRDC outbreaks. In this work, the most frequent coinfections detected were BVDV and PIV-3 in 8% of the samples, with the highest prevalence (12%) found in River Nile State. These results are unlike those (PPR and BVDV) reported in camel lungs in Sudan (Intisar et al. 2015a). Our results are higher than variable reports from different countries; for example, antibodies confirming coinfection of BVDV and PIV-3 were detected in 0.4% of samples from dairy cattle in Brazil (Yoshitani et al. 2024). In India, coinfection of BPIV3 and BRSV was found in 6% of samples (Yadav et al. 2024), in Egypt, coinfection of BRSV and PIV-3 was detected in 5% of examined samples (Zaher et al. 2014). In Mongolia, coinfection positive results were found in 9% for BVDV and BHV-1, and 4% for BPIV3 and BRSV (Guo et al. 2021). Coinfection of BVDV and RSV in our study was observed in only two samples in River Nile State (1%); however, it was also higher than other reports elsewhere. In Brazil, antibodies to both BVDV and RSV were found in 0.2% of samples (Yoshitani et al. 2024); another study detected associations between BVDV + BRSV, BVDV + BRSV + bovine coronavirus (BCoV) (De Oliveira et al. 2022). Existence of the three viruses, BVD, PIV-3 and RSV was detected in two samples (1%) in this study, also in River Nile State, which is lower than those detected (10%) in Brazil (Yoshitani et al. 2024). In Spain, the existence of BVDV, BRSV, BCoV and PIV-3 associated with BRD was found in 40% – 72% of outbreaks (Bernal et al. 2023); in China, coinfections of BVDV, BRSV, Bovine Herpes Virus Type 1 (BoHV-1) and BPIV-3 were detected in 50% of cases (Zhou et al. 2023) and in Colombia, evidence for coinfection of BVDV, BRSV and BPIV-3 was found through detection of its antibodies in all examined cattle herds (León et al. 2019). Also in Saudi Arabia, antibodies to BHV-1 + BVD, + bovine adenovirus-3 (BAdV-3), to BHV, BHV-1 + RSV, to BVD-1 + BVD + RSV + BPI3 (bovine parainfluenza 3) and to BHV-1 + BVD + RSV + BPI3 were detected (Ali & Gomaa 2014). In Columbian buffaloes, seropositivity of BVD, RSV, BPI3 and BHV-1 coinfection was 32% to two viruses, 29% to three viruses, and 7% to the four viruses (Pastrana et al. 2022). In Kenya, positive associations between Infectious bovine rhinotracheitis (IBR), PIV3 and BVDV in calves were confirmed (Callaby et al. 2016).

## Conclusion

The results of this study confirmed the previous reports about the presence of BVDV, PIV-3 and RSV infections in cattle in Sudan, beside the determination of coinfections with these viruses for the first time. Detailed epidemiological and molecular studies for the investigation and molecular characterisation of these viruses, as well as other respiratory viruses in different areas of Sudan, are highly recommended to aid in the control of these economically significant viral infections.
